# Quantitative Epigenetics: A New Avenue for Crop Improvement

**DOI:** 10.3390/epigenomes4040025

**Published:** 2020-11-07

**Authors:** Vijay Gahlaut, Gaurav Zinta, Vandana Jaiswal, Sanjay Kumar

**Affiliations:** 1 Biotechnology Division, CSIR-Institute of Himalayan Bioresource Technology, Palampur, Himachal Pradesh 176061, India; gzinta@gmail.com (G.Z.); sanjaykumar@ihbt.res.in (S.K.); 2 Academy of Scientific and Innovative Research (AcSIR), CSIR-IHBT, Palampur, Himachal Pradesh 176061, India

**Keywords:** DNA methylation, epialleles, epiRILs, epigenetics, epigenome-wide association studies

## Abstract

Plant breeding conventionally depends on genetic variability available in a species to improve a particular trait in the crop. However, epigenetic diversity may provide an additional tier of variation. The recent advent of epigenome technologies has elucidated the role of epigenetic variation in shaping phenotype. Furthermore, the development of epigenetic recombinant inbred lines (epi-RILs) in model species such as Arabidopsis has enabled accurate genetic analysis of epigenetic variation. Subsequently, mapping of epigenetic quantitative trait loci (epiQTL) allowed association between epialleles and phenotypic traits. Likewise, epigenome-wide association study (EWAS) and epi-genotyping by sequencing (epi-GBS) have revolutionized the field of epigenetics research in plants. Thus, quantitative epigenetics provides ample opportunities to dissect the role of epigenetic variation in trait regulation, which can be eventually utilized in crop improvement programs. Moreover, locus-specific manipulation of DNA methylation by epigenome-editing tools such as clustered regularly interspaced short palindromic repeats/CRISPR-associated protein 9 (CRISPR/Cas9) can potentially facilitate epigenetic based molecular breeding of important crop plants.

## 1. Introduction

Epigenetic modifications modulate gene expression without any change in genomic DNA sequences that affects multiple aspects of plant growth and development [1]. These epigenetic modifications mainly involve DNA methylation, histone modification, and small RNA (sRNA)-mediated modifications [2]. Of these epigenetic modifications, DNA methylation is relatively well studied. In plants, DNA methylation predominantly occurs in the cytosines (C) of three sequence contexts: CpG, CpHpG, and CpHpH, where H represents any base other than G (i.e., A/C/T). DNA methylation at each sequence context is regulated by a particular set of enzymes named cytosine-5 DNA methyltransferases (C5-MTases) having complementary ‘de novo’ and ‘maintenance’ methylation activities [3,4]. In the de novo methylation process, unmethylated cytosine residues are methylated, while in methylation maintenance the preexisting methylation patterns are maintained after DNA replication [5]. Different C5-MTases including Domains Rearranged Methylases (DRMs), Methyltransferases (METs), and Chromomethylases (CMTs) participate in these processes. The DRMs are involved in de novo DNA methylation via RNA-directed DNA methylation (RdDM) in all three DNA sequence contexts [6]. The DRM2, an ortholog of mammalian DNMT3, is involved in CpHpH methylation of euchromatic regions [7]. The METs are involved in the maintenance of CpG methylation during DNA replication [8]. The CMTs are involved in the maintenance of CpHpG and CpHpH methylations. In *Arabidopsis thaliana* (L.) Heynh (Arabidopsis), CMT2 catalyzes CpHpH methylation, while CpHpG methylation is catalyzed by CMT3 and to a lesser extent by CMT2 [9,10]. The roles of DNA methylation in plant development and responses to environmental stress conditions have been discussed in detail previously [11].

The recent advances in bisulphite sequencing (BS-Seq) and methylC-sequencing (MethylC-seq) allow profiling of DNA methylation status across entire genomes within a species [8,12]. Also, species-level epigenomic diversity in the natural populations of Arabidopsis collected from diverse locations of the globe has been determined [13]. Whole-genome bisulfite sequencing (WGBS) is particularly powerful as it constructs the genomic maps of DNA methylation at a single-base resolution level [14]. WGBS has been utilized in the methylome analysis of several plant species including model plants Arabidopsis and crop plants *Zea mays* and *Triticum aestivum* [15,16,17]. WGBS analysis in Arabidopsis has shown that 5.26% of all genomic C bases are methylated and their allocation on the genome was uneven, for instance, about 24% of CpG context was found to be methylated, which is followed by CpHpG (6.70%), and CpHpH (1.70%) contexts [15,18]. However, substantial variation in methylation patterns has been observed between plant species (see Table 1). For instance, CpG context methylation ranged from 24% (Arabidopsis) to 93% (*Cicer arietinum*), CpHpG methylation varied from 3.48% (*Triticum aestivum*) to 89 % (*Cicer arietinum*), and CpHpH methylation ranged from 1.36% (*Eucalyptus grandis*) to 38% (*Cicer arietinum*). The CpG methylation is more prevalent and makes the largest portion of total DNA methylation in plants. Many factors determine the observed variation in methylation levels in different contexts, which include genome size, architecture, and distinction in the activity of methylation targeting pathways.

DNA methylation together with histone modifications and non-histone proteins delineates chromatin structure and its accessibility to transcriptional machinery. Thus, it plays an important role in gene expression regulation, transposon element (TE) silencing, and trait inheritance [24]. In plants, DNA methylation levels are generally higher at TEs as compared to genic regions. Silencing TEs is required to maintain genome stability, and it is mediated via RdDM [25]. In spite of having a crucial role in distinct biological processes, the application of DNA methylation in crop improvement remains to be fully explored. The epialleles (epigenetic alleles) are loci that differ in chromatin states and get transmitted to the next generations [26,27,28]. Epialleles provide an additional source of variation, which are involved in the regulation of phenotypic diversity. Various stable epialleles affecting floral morphology [29], flowering time [30], disease resistance [31], pigmentation [32], and leaf senescence [33] have been reported in different plant species. In Arabidopsis, epigenetic recombinant inbred lines (epiRILs) have been developed, which show variation and high heritability for traits like flowering time and plant height as well as stable inheritance of DNA methylation variants [34]. Utilizing this epiRIL population, epigenetic quantitative trait loci (epiQTL) controlling flowering time and primary root length were identified that showed high heritability (up to 90%) [35]. Considering that some epiQTLs are stably inherited and show phenotypic diversity, they are good targets for natural/artificial selection for crop improvement.

In this review, we first discuss various epialleles controlling phenotypic traits in plants. Second, the development of epiRILs and epiQTL mapping populations in Arabidopsis and other important crop plants are described, which can be used for quantitative epigenetic studies to identify epigenetic variants controlling trait. Finally, locus-specific manipulation of DNA methylation levels by using site-specific nucleases to generate epialleles is highlighted, which can be utilized for crop trait improvement.

## 2. Epialleles (Natural and Mutagen Induced)

Although a number of genes/QTLs have been identified in various plants, however, missing heritability is still a major challenge for researchers and breeders where unknown components regulate phenotype in addition to genes/QTLs. Epigenetic modifications could be one of the major causes of missing heritability [36]. Changes in the DNA methylation status of a particular gene may affect its expression and can be trans-generationally inherited, which leads to trait variation [37]. Such stably inherited epigenetic variants are referred to as epialleles that contribute to phenotypic variation in plants.

Several epialleles have been reported in the model plant Arabidopsis and crop plants like rice, maize, field mustard (details are given in Table 2). The first classical example of epiallele was reported in Arabidopsis and known as *clark-kent* (*clk*). It is a natural epimutant with an enhanced number of stamens and carpels. In this epimutant, hypermethylation of cytosine occurred at flower development locus *SUPERMAN* [29]; while hypomethylation of this locus in *clk* mutants was found unstable and reverted to the wild type phenotype. A total of seven independent *clk* mutants were reported with similar phenotypes. Peloric mutant is another classical example of epialleles found in toadflax (*Linaria vulgaris*). Peloric mutants (radial flower) showed different flower symmetry as compared to wild type plants (bilateral flower). In this epimutant, hypermethylation occurs at the promoter of *Lcyc* gene (homolog of *CYCLOIDEA* gene of Antirrhinum, responsible for floral symmetry) and led to the silencing of the gene. This epimutation is stably inherited across the generation over 100 years; however, loss of hypermethylation of the promoter of the Lcyc gene may regain bilateral floral symmetry of wild type [38]. In tomato, two epialleles were reported that affect fruit ripening and vitamin E accumulation. Hypermethylation at the promoter of *colorless non-ripening* (*cnr*) locus encoding SBP-box transcription factor (TF) causes ripening defective fruits in tomato [39]. Similarly, DNA methylation of *Vitamin E* (*VTE3*) gene promotor affects its expression that leads to vitamin E accumulation in tomato fruits [40]. In melon, it was shown that DNA hypermethylation in the promoter of *CmWIP1* (*WASP/N-WASP-interacting protein 1*) TF causes the transition from male to female flowers [41]. In the case of rice, six epialleles were reported that affect phenotypes such as dwarf stature, panicle architecture, leaf angle, seed size, and photosynthetic capacity (Table 2). In Brassica, the epiallele *S locus protein 11/S locus* (*SP11/SCR* locus) was involved in the dominance effect that regulates the self-incompatibility [42]. 

Another important epigenetic phenomenon is known as paramutation. It was initially observed in the *r1* (*red1*) locus that regulates anthocyanin pigment biosynthetic pathway in maize [49], and subsequently found in *booster1* (*b1*), *purple plant1* (*pl1*), and *pericarp color1* (*p1*) loci, which also regulate anthocyanin pigment biosynthetic pathway [32,50,51], and *low phytic acid1* (*lpa1-241*) that was involved in phytic acid biosynthesis [52]. Besides spontaneous mutations, small interfering RNA (siRNA) also plays a vital role in the development of epialleles in plants. In Arabidopsis, silencing of the *folate transporter 1* (*AtFOLT1*) gene is regulated by siRNAs derived from the truncated copies of *AtFOLT2* locus, which causes reduced fertility [46]. Furthermore, methylation of transposable elements is also found to affect the phenotype substantially. For instance, hypomethylation of intronic TE (Karma retro TE) caused abnormal splicing of *DEFICIENS* (*DEF*) gene, resulting in parthenocarpy and reduced yield in oil palm [59]. Moreover, epialleles are well known to control manytraits including flower/fruit-related traits, sex determination, plant architecture and vitamin accumulation in different plant species (see Table 2). Epialleles also regulate the homeostasis between euchromatin and heterochromatin to maintain genome stability, as the loss of heterochromatin would expose genes to DNA methylation machinery. It was recently, shown that DNA methylation is inversely correlated to heterochromatin in Arabidopsis [60]. 

## 3. Epigenetic Recombinant Inbred Lines (epiRILs)

EpiRILs are referred to as the recombinant inbred lines that differ for DNA methylation patterns and show no genetic variation. EpiRILs represent an excellent resource to identify the effect of DNA-methylation on phenotypes [34]. Under changing climatic conditions, epigenetic modifications could play a crucial role in plant adaptation to environmental stresses [61]. epiRILs developed in Arabidopsis have been used to explore the effect of environmental factors, which revealed that stress-induced epigenetic modifications could be heritable and provide phenotypic plasticity to plants to endure stress [62].

**Figure 1:**
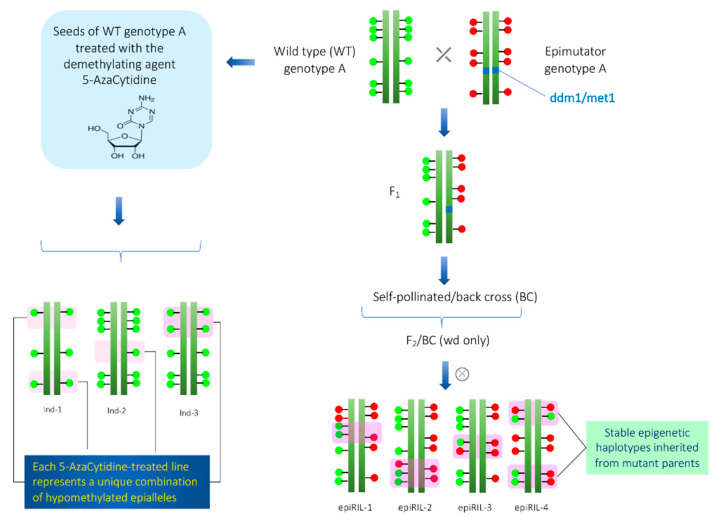
Construction of epiRILs (hypomethylated population) for quantitative epigenetics. In the left side, the scheme for the construction of hypomethylated population using demethylating agent 5-AzaCytidine is shown. On the right side, scheme for construction of epiRILs with stable inheritance by crossing of two parents (wild-type and epimutator parents (*met1* or *ddm1*)) with different epigenetic states is shown. The green and red circles that overlay the genome sequence illustrates the different epigenetic states of the two parents.

In Arabidopsis, at least two different epi-RIL populations have been developed [34,63]. One epi-RIL population was derived from the crossing of *met1* mutant and its isogenic wild type [63]. The *met1* mutant is defective in DNA methyltransferase [8,64]. In the F_2_ and subsequent generations, only wild type *MET1* alleles were selected in order to prevent de novo DNA methylations. In each generation, progenies were advanced using the single seed descent method. Similarly, another epi-RIL population was derived from the crossing of *ddm* mutant and its isogenic wild type [34]. The *ddm* mutant is defective in the *DDM* locus that encodes nucleosome-remodeling ATPase required to maintain C methylation [65,66]. Due to the utilization of isogenic lines in crossing, these epiRILs have no genetic variations; however, epigenetic variations are maximum. Schematic representation of the development of epiRILs is given in Figure 1. The following points need to be considered while studying transgenerational epigenetic variations.

### 3.1. Persistence of Epigenetic Modification in the epiRILs

Epigenetic modifications are heritable and maintained across several generations as revealed from epiRILs developed by the crossing of mutants (*met1* or *ddm1*) with its isogenic wild types [67,68]. In the *ddm* epiRILs, epigenetic loci targeted by small RNAs were extensively remethylated, while nontargeted loci remained unmethylated [69]. In contrast, *Flowering Wageningen* (*FWA*) epiallele associated with flowering time became methylated in the subsequent generations despite being targeted by small RNA [34,63]. The mechanism underlying remethylation process and the role of small RNAs in remethylation remain unclear. However, a recent study provides mechanistic insights on the formation and transmission of epialleles and suggested that histone and DNA methylation marks are critical in determining the ability of RdDM target loci to form stable epialleles [70]. High levels of active histone mark H3K4me3 at specific loci prevent recruitment of the RdDM machinery, whereas high levels of H3K18ac enable ROS1 (Repressor of Silencing 1) to access specific loci, therefore antagonizing RdDM-mediated re-establishment of DNA methylation.

### 3.2. Phenotypic Variation and Stability in the epiRILs

Continuous variations for different traits observed in the abovementioned two epi-RILs suggested that epigenetic modification was involved in the regulation of polygenic traits also. Although two epi-RILs differed for different traits. For instance, epiRILs derived from *met1* mutant showed variation for biotic (bacterial pathogen) and abiotic stress (salt) tolerance [63]; however, in the case of epiRILs derived from *ddm1* mutant, variation can be clearly seen for morphological traits like flowering time and plant height [34]. 

The stability of phenotypic characters in the above two epiRILs is quite different. Phenotypes in *met1* derived epiRILs are very unstable, and several lines were unable to advance to F_8_ generation due to abnormal development and infertility [63]. Unlike, *ddm1* derived epiRILs were found highly stable and more than 99% lines advanced to F8 generations without any abnormality [34]. 

To explain phenotypic instability, Reinder et al. [63] studied the methylation pattern in *met1*-derived epiRILs. They found that some cytosine methylation sites were highly segregating even in F_8_/F_9_ generations and suggested that some methylations sites are very unstable and cannot be fixed by repeated selfing. Furthermore, several ectopic and hypomethylations were observed that were different from any of the parental genotypes, suggesting de-novo methylations may be a possible reason for the phenotypic and epigenetic instability across generations in *met1* derived epiRILs [63]. In contrast, in *ddm1*-derived epiRILs, cytosine remethylation seems to be the reason for nonparental cytosine methylation [69].

### 3.3. Epigenetic Basis of Heterosis

Heterosis is a phenomenon where F_1_ hybrid shows enhanced phenotypes than their respective parents. Heterosis has extensively been exploited as a potential breeding strategy for crop improvement. The importance of heterosis, its application in plant breeding, and molecular mechanisms underlying heterosis have been discussed elsewhere [71,72,73,74]. Many genetic factors contribute to the heterotic phenotype; however, epigenetic interactions between two parental alleles also play a critical role in heterosis [75,76]. Hybrids of Arabidopsis derived from two genetically similar but epigenetically diverse ecotypes C24 and Ler showed more than 250% enhanced biomass over parents advocated the importance of epigenetics in heterosis [77]. Out of three different cytosine methylation contexts, the highest alteration was observed at CpG site; however, CpHpH and CpHpG methylations slightly increased in hybrids than parental genotypes [75]. Furthermore, 75% methylation changes were observed in TEs, and more than 95% methylation increase was observed in siRNA generating regions [75]. In Arabidopsis, hybrids derived from Col-wt (female parent) and 19 epiRILs (male parents) showed positive and negative heterosis for six growth-related traits. DNA methylation profiling of hybrids and parents identified epiQTLs associated with heterosis and suggested that DMRs present in the parental genotypes are responsible for heterosis [74]. The above results provide evidence that epigenetic components act as critical determinants of heterosis, which can be exploited for crop improvement. 

## 4. Development of Epigenetically Modified Population by Chemical Agents

The generation of epigenetic mutants like *met1* or *ddm1* is still not very common in non-model plants. Therefore, chemical agents can be used to induce epigenetic modifications that may serve the purpose to study the effect of epigenetics on quantitative traits. Several chemical compounds are known to induce epigenetic modifications—including DNA methylation, histone modification, etc.—with different modes of action [78]. For instance, chemicals like 5-Azacytidine (5-AzaC), 5-Aza 2′deoxycytidine, and Zebularine inhibit the methyltransferase activity and lead to the reduction of transfers the methyl group from S-adenosylmethionine (SAM) to the cytosine ring. Mutagenesis with these chemicals would lead to the development of hypomethylated population. On the other hand, some chemicals act as histone deacetylase inhibitors and increase the histone acetylation and reactivate the silent genes. Chemicals like trichostatin-A (TSA), Helminthosporium carbonum (HC) toxin, nicotinamide, diallyl disulfide sodium butyrate etc. are good examples of histone deacetylase inhibitors. Besides these, some chemicals—like sulfamethazine (SMZ), ethionine, and dihydroxypropyl adenine (DHPA)—hamper the supply of methyl groups. These chemicals decrease the folate pool and cause methyl deficiency. 

The availability of a wide range of chemical mutagens would be a great asset to create epigenetically modified populations in plants. A successful example of the adoption of chemical methods to develop a hypomethylated population in Brassica, where epialleles were developed by using 5-AzaC, and this hypomethylated population showed variability for different phenotypic traits [79].

Transgenerational inheritance of some phenotypes has also been demonstrated. When compared to untreated controls, BraRoAZ population (hypomethylated population of *B. rapa* line R-o-18) showed a decrease immuno-staining of 5mC on pachytene chromosomes. Furthermore, methylation sensitive amplified polymorphism (MSAP) showed high divergence as well as variability. High phenotypic variability was also observed for different seed related characters like yield, protein content, oil content, erucic acid, linoleic acid, and palmitic acid. Each line in the BraRoAZ population represented a unique combination of hypomethylated epialleles. Thus, efforts should be made for the development of epigenetically modified populations using chemical agents in other crops. 

## 5. Development of User-Friendly Epigenetic Markers 

Epigenetic markers are important to study quantitative epigenetics and identify epialleles associated with traits of interest. Several epigenetic markers have been identified to be involved in biological and molecular functions in plants [36,80,81,82]. However, utilization of these methylation marks into crop breeding programs is still lacking. Developing a cost-effective and easy genotyping platform for identifying and selecting desirable epiallele is needed in crop plants. For the identification of epialleles, three major approaches are available and widely used in the case of human (i) bi-sulfite sequencing PCR (BSP), (ii) methylation-specific PCR (MSP), and (iii) chop-PCR [83,84]. The first two approaches require bi-sulfite conversion of non-methylated cytosine to uracil. Bisulfite conversion is a very important and crucial step in these methods. Classical bisulfite conversion approaches involve lots of effort and time, even though conversion efficiency and yield are less. With advancement in technologies, now commercial kits are available with high yield and more than 99% conversion efficiency [85]. These kits also facilitate the rapid conversion within 3–4 h. An exhaustive comparison of different commercially available kits for bisulfite conversion efficiency and cost-effectiveness has been published elsewhere [86]. In BSP, after bisulfite conversion, the targeted region is PCR amplified and then sequenced to identify the epialleles. However, in MSP, sequencing is not required, and the targeted region is amplified with methylation-specific primer pairs to distinguish epialleles. In chop-PCR, genomic DNA is partially digested with methylation-sensitive restriction enzymes (MSREs) followed by PCR amplification of the targeted region. Furthermore, quantitative measurement of DNA methylation can be done through MethylLight and methylation-sensitive high-resolution melting (MS-HRM). 

Although the development and utilization of epigenetic markers are very much successful in humans [87], however, this area of research lags behind in the case of plants. Therefore, there is an urgent need to develop online platforms and databases to assess f of epigenetic markers with full description. 

## 6. Quantitative Epigenetic Models for Complex Trait

Several statistical methods exist to detect epigenetic variations and their impact on the phenotype or epiQTLs [36,80,81,82]. The significance and accuracy of epiQTLs identification are affected by several factors like recombination, transgressive segregation, instability of epialleles, and parent off origin effect. These factors may create confounding effects during epiQTLs analysis and result in false positives or false negatives. To deal with these interrupting factors, Johannes and Colome-Tatche [81] suggested that population derived from crossing between isogenic lines (dissimilar for epigenetic marks) are the most suitable material. Tal et al. [80] derived covariances between kinship due to epigenetic transmissibility and environmental effect. They modeled the number of chances for epigenetic reset between generations and environmental induction, and estimated the heritable epigenetic variance and epigenetic transmissibility. Furthermore, multiple testing is the major drawback of quantitative genetics because it can give several false positives. Jaffe et al. [88] developed a statistical model to deal with multiple testing corrections during genome-wide identification of epigenetic variability. These models can be useful to study the missing heritability contributed by epigenetic variation [81,89], but did not consider the phenotypic variation contributed by epigenetic variation. Wang et al. [90] suggested a model to estimate phenotypic variation explained by epigenetic variation and their effects on phenotypic values and also the interaction of epigenetic and genetic effects (additive and dominant). This model also predicts the proportion of genetic variation contributed by epigenetic modifications.

## 7. EpiQTLs and Epigenome-Wide Association Study (EWAS)

As discussed, epigenetic markers are stably inherited across generations and are randomly present in the genome with high frequency. These features allow the exploitation of epigenetic markers in the identification of epiQTLs. Unlike QTLs, epiQTLs are epigenomic loci where no polymorphism for the DNA nucleotide sequence occurs but they differ for cytosine methylation levels, and these differential methylation patterns regulate phenotypic variation of quantitative traits. In Arabidopsis, Cortijo et al. [35] identified major epiQTLs explaining 60–90% heritability for quantitative traits like root length and flowering time using *ddm1*-derived epi-RILs. These epiQTLs were found reproducible and useful for artificial selection. Furthermore, 99.9% epialleles were found stable; and based on the inheritance and recombination events epigenotype map (E-map) was constructed using mutagenic accumulation lines [26]. Another study in Brassica identified epiQTLs for seven agronomic traits using methylation-sensitive amplified fragment length polymorphism (MS-AFLP) and retrotransposon epimarkers [91]. High stability of epigenetic marks was found at different developmental stages, environmental conditions and transgenerational levels. In Sorghum, by implementing MSAP genotyping approach, 122 methylation polymorphic loci were generated to construct E-map, which harbored methylation hotspots [92]. In soybean, co-segregation of differentially methylated regions (DMRs) in RILs allowed the identification of methylQTL (QTLs associated with DNA methylation) [93]. Thus, the stable inheritance of epialleles across generations makes it a potential regulator of phenotypic variations in crops where genetic variation is negligible [26].

**Figure 2:**
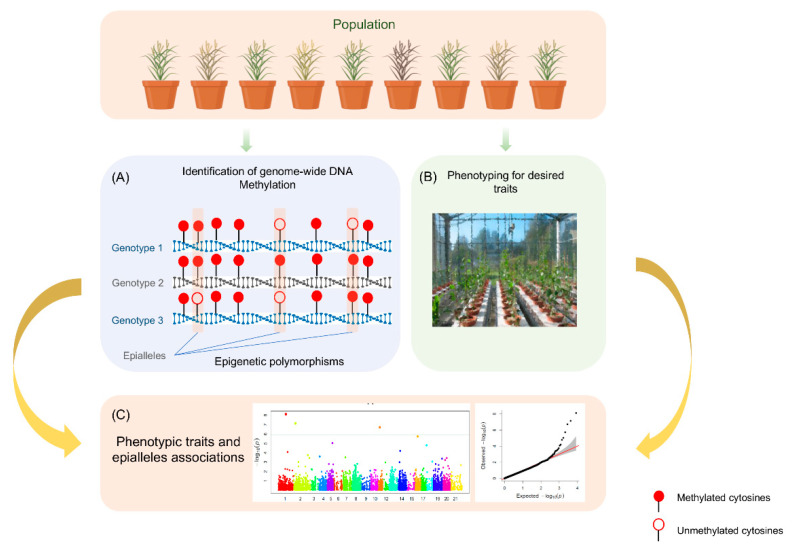
Schematic representation of epigenome-wide association mapping (EWAS) involving three major steps: (A) epigenotyping to explore different epialleles, (B) precise phenotyping of diverse germplasm, (C) statistical analysis to identify EpiQTLs.

Like genome-wide association studies (GWAS), epigenome-wide association study (EWAS) may prove a worthwhile approach to explore the impact of the epigenetic modifications on phenotype where genome-wide epimarkers are available (Figure 2). EWAS is based on linkage disequilibrium (LD) mapping, which utilizes natural populations and historic recombination events and can thus accomplish high-resolution mapping. A number of EWAS studies have been conducted in humans, which identified that epigenetic modifications are associated with several human diseases like Parkinson’s disease [94], coronary artery disease [95], Alzheimer’s disease [96], and Type 2 diabetes [97]. Moreover, to gather the extensive knowledge generated through EWAS in humans, EWAS Atlas has also been developed [98]. However, there is a limited number of EWAS performed in plants, and so far, a single EWAS study identified epigenetic modification associated with a mantled abnormality in oil palm [59]. By using somatic clones (diverse for mantled abnormality and oil yield), a locus MANTLED was identified where hypomethylation in LINE retrotransposon leads the alternate splicing and premature termination.

Linkage mapping and LD mapping have their own importance and limitations. To overcome limitations, integrated genetic mapping by combining linkage and linkage-disequilibrium is recommended. Recently, an integrated linkage and linkage-disequilibrium mapping was conducted for the identification of epiQTLs in plants [99]. Using 550 F_1_s and 435 natural germplasm accessions, manyepiQTLs (163 epiQTLs) were found to be associated with growth and wood properties in Populus. Furthermore, 23 causal genes present within epiQTL regions showed cause and effect relationship as revealed by the coregulation of eQTN (expression quantitative trait nucleotide) and eQTM (expression quantitative trait methylation) [99].

Identification of epiQTLs by using any of the methods mentioned above, high-density epi-genotyping is a significant challenge. However, with the advancement in sequencing technologies, high-density epi-genotyping is now possible. Different techniques like epigenetic based restriction-site associated DNA sequencing (epiRADseq), bisulfite-converted restriction site-associated DNA sequencing (bsRADseq), epi-genotyping by sequencing (epi-GBS) [100,101,102] are available for high-density epi-genotyping. Each of these methods has its advantages and limitations. For instance, epiRADseq is as cheap as compared to bsRADseq; however, it utilizes *HpaII* methylation-sensitive restriction enzyme, thus it is restricted to only limited sites. The bsRADseq covers all the context of cytosine methylations, but it requires a reference genome for downstream analysis, therefore it is not suitable for non-reference based organisms. Epi-GBS is a cost-effective and less time-consuming approach. Although the cost of epi-GBS can further be reduced by using single hemi-methylated adapters [103]. Moreover, Epi-GBS can also be applied to plants where a reference genome is not available. A successful example of utilization epi-GBS for identification of DMRs is available in the case of almond where epi-GBS identified more than 7000 methylated fragments and among these 677 fragments were differentially methylated. Furthermore, the methylation state of some gene-coding sequences was found to be associated with flower bud dormancy [104].

As mentioned earlier, several epialleles have been reported in plants, and huge opportunities still exist to explore it further in various plant species. Thus, we strongly recommend that EWAS should be performed in crop plants, which will contribute to improving our understanding of epigenetic mechanisms regulating phenotypic traits, and also expedite crop improvement programs through epibreeding.

## 8. Epigenome Editing Using Site-Specific Nucleases

Several tools are reported that allow site-specific manipulation of DNA methylation/demethylation using programmable DNA-binding proteins [zinc finger (ZFs) proteins and CRISPR-dCas9] in plants [105]. In Arabidopsis, it was shown that ZF fused with RdDM component SU(VAR)3-9 HOMOLOG 9 (SUVH9) was able to cause target methylation to the *FWA* promoter. It caused *FWA* silencing via heritable methylation and led to the late-flowering phenotype [106]. Recently, Gallego-Bartolome et al. [107] tested the capability of several other RdDM components (i.e., RNA-dependent RNA polymerase 2 (RDR2), Sawadee Homeodomain Homolog 1 (SHH1), Microrchidia 1 (MORC1), MORC6, Defective in Meristem Silencing 3 (DMS3), and RNA-Directed DNA Methylation 1 (RDM1)) to promote targeted DNA methylation at *FWA* locus when fused with ZF. This study provides a theoretical framework that can be utilized to design efficient targeted DNA-methylation programs in plants. In addition to ZF nucleases, CRISPR-dCas9 was also used to target DNA methylation in plants [108]. Recently developed CRISPR-Cas9-SunTag system having the catalytic domain of tobacco DRM has been used to target a *FWA* locus that showed heritable DNA methylation in Arabidopsis [108]. Besides DNA methylation, targeted DNA demethylation has also been performed by fusing Ten-Eleven Translocation 1 (TET1) to both ZF and CRISPR-Cas9-SunTag systems in Arabidopsis [109]. The DNA demethylation achieved at the *FWA* promoter was found highly specific and heritable in nature. Further development of these tools for targeted DNA methylation and demethylation in plants other than Arabidopsis will open up new avenues to study locus-specific effects of DNA methylation and could be used for the generation of new epialleles.

Epigenome editing techniques utilizing CRISPR system have significant potential for crop improvement. However, in a recent international survey it has been found that consumers equate CRISPR/Cas to traditional genetically modified organisms (GMO) [110]. Notably, CRISPR technique introduce changes to DNA intrinsic to the target species, while traditional genetic modification introduces foreign DNA from a different species (i.e., transgenic) or another cultivar of the same species (i.e., cisgenic) [111]. Importantly, DNA-free delivery of CRISPR-Cas9 ribonucleoproteins might be considered non-GM crops. This would open the door for the development and commercialization of superior crops in various countries even where GM crops are unacceptable.

## 9. Conclusions and Future Prospects

Strengthening crop improvement programs is crucial to feed the global population. Utilization of epigenetic information at the epiQTLs and epialleles level may provide new prospects for crop improvement as existing breeding methods primarily focus on genetic and ignore epigenetic aspects. The advancement of new sequencing technologies like BS-seq (bisulphite sequencing) and MethylC-seq (methylC-sequencing) can help to delineate the epigenetic basis of trait determination. This information will eventually enhance the inclusion of epigenetic methods in crop improvement. Furthermore, loci-specific DNA methylation patterns can be achieved in plant genomes by fusion of catalytic domains of de novo DNA methylation or demethylation enzymes with nucleases (i.e., zinc finger nucleases (ZFNs), and clustered regularly interspaced short palindromic repeats/dCRISPR-associated protein 9 (CRISPR–dCas9 systems)). These systems have been employed to engineer epigenomes of mammalian cell lines [112,113] and plants [108,109]. Epigenome engineering will not only help in the functional validation of DNA methylation patterns regulating phenotypic traits but will also help to generate desirable traits by creating epigenomic diversity and can accelerate crop improvement by epibreeding [106].

## Figures and Tables

**Table 1:** Genome size, total number of repeat elements, DNA methylation levels at three sequence contexts (CpG, CpHpG, and CpHpH) in different plant species, where methylation % indicates per-site methylation

Species (Common Name)	Family	Monocot/Eudicot	Genome Size (Mb)	Repeat Elements	CpG (%)	CpHpG (%)	CpHpH (%)	Reference
*Arabidopsis thaliana* (Arabidopsis)	Brassicaceae	Eudicot	135	31,189	24.00	6.70	1.70	[15]
*Beta vulgaris* (beet)	Amaranthaaceae	Eudicot	758	656,014	92.00	81.00	18.80	[4]
*Brassica oleracea* (cabbage)	Brassicaceae	Eudicot	648	532,987	52.50	22.00	5.11	[4]
*Brassica rapa* (mustard)	Brassicaceae	Eudicot	485	218,781	37.20	17.28	4.44	[4]
*Cajanus cajan* (pigeonpea)	Fabaceae	Eudicot	833	1,127,729	70.23	54.60	9.87	[19]
*Camellia sinensis* (tea)	Theaceae	Eudicot	3100	5,164,785	82.00	70.00	10.00	[20]
*Cannabis sativa* (canabis)	Cannabaceae	Eudicot	818	376,401	75.50	65.00	8.72	[4]
*Capsella rubella* (pink shepherd’s purse)	Brassicaceae	Eudicot	219	39,716	32.00	9.90	34.70	[4]
*Cicer arietinum* (chickpea)	Fabaceae	Eudicot	738	853,514	93.00	89.00	38.00	[21]
*Citrus clementina* (clementine)	Rutaceae	Eudicot	370	205,699	45.83	25.13	8.26	[4]
*Cucumis sativus* (cucumber)	Cucurbitaceae	Eudicot	367	57,750	45.88	16.50	4.12	[4]
*Eucalyptus grandis* (rose gum)	Myrtaceae	Eudicot	640	689,306	37.12	19.96	1.36	[4]
*Fragaria vesca* (strawberry)	Rosaceae	Eudicot	240	129,500	48.35	20.63	2.32	[4]
*Glycine max* (soybean)	Fabaceae	Eudicot	1115	38,581	63.20	38.40	4.10	[22]
*Gossypium raimondii* (cotton)	Malvaceae	Eudicot	880	489,564	71.97	57.80	13.14	[4]
*Lotus japonicus* (birdsfoot trefoil)	Fabaceae	Eudicot	472	160,505	67.75	36.59	8.66	[4]
*Malus domestica* (apple)	Rosaceae	Eudicot	742	1,245,768	63.50	44.14	4.57	[4]
*Manihot esculenta* (cassava)	Euphorbiaceae	Eudicot	742	258,416	51.53	30.38	1.90	[4]
*Medicago truncatula* (barrel clover)	Fabaceae	Eudicot	465	375,003	59.80	16.94	5.09	[4]
*Populus trichocarpa* (poplar)	Salicaceae	Eudicot	500	173,230	43.95	26.78	5.01	[4]
*Prunus persica* (peach)	Rosaceae	Eudicot	265	95,678	50.18	19.59	3.64	[4]
*Ricinus communis* (castor bean)	Euphorbiaceae	Eudicot	323	575,449	64.54	37.94	11.97	[4]
*Solanaceae lycopersicum* (tomato)	Solanaceae	Eudicot	907	887,009	84.05	54.84	8.35	[4]
*Solanaceae tuberosum* (potato)	Solanaceae	Eudicot	840	404,861	70.90	42.20	15.80	[23]
*Vitis vinifera* (grape vine)	Vitaceae	Eudicot	487	449,466	45.95	20.43	1.15	[4]
*Brachypodium distachyon* (stiff brome)	Poaceae	Monocot	352	51,793	49.17	19.17	1.41	[4]
*Oryza sativa* (rice)	Poaceae	Monocot	430	447,163	54.70	37.30	12.00	[16]
*Panicum hallii* (Hall’s panicgrass)	Poaceae	Monocot	550	154,970	56.28	29.97	2.43	[4]
*Panicum virgatum* (switchgrass)	Poaceae	Monocot	1600	1,793,620	53.56	35.74	3.06	[4]
*Setaria viridis* (green foxtail)	Poaceae	Monocot	515	372,068	44.49	23.25	1.56	[4]
*Sorghum bicolor* (sorghum)	Poaceae	Monocot	730	397,003	84.75	73.25	5.81	[4]
*Triticum aestivum* (wheat)	Poaceae	Monocot	17,000	3,968,974	53.30	3.48	1.41	[17]
*Zea mays* (maize)	Poaceae	Monocot	2665	1,971,471	86.00	74.00	5.40	[7]

**Table 2:** List of some stable epialleles reported in different plant species

Species	Gene/Locus	Epigenetic Variation	Phenotypic Traits	References
*Arabidopsis thaliana*	*SUP (SUPERMAN)*	Mutagen induced	Increased numbers of stamens and carpels	[29]
	*FWA* (Flowering Wageningen)	Mutagen induced	Late flowering	[30]
	*PAI2* (Phosphoribosyl Anthranilate Isomerise)	Trans-acting (small RNAs)	Only gene expression affected; no specific phenotype	[43]
	*BAL1*	Mutagen induced	Dwarfing and elevated disease resistance	[31]
	*AG (AGAMOUS)*	Mutagen induced	Affect flower structure	[44]
	*BNS (BONSAI*)	*ddm1*-induced syndrome	Stunted growth	[45]
	*FOLT1(folate transporter 1)*	Trans-acting (small RNAs)	Reduced fertility	[46]
	*QQS (Qua-Quine Starch*)	Spontaneous	Higher starch accumulation	[47]
	*PPH (Pheophytin Pheophorbide Hydrolase)*	Spontaneous	Inhibits leaf senescence	[33]
	*HISN6B* *(Histidinol-phosphate aminotransferase 1)*	Spontaneous	Hybrid incompatibility	[48]
*Zea Mays*	*r1* (red1)	Spontaneous	Reduced pigmentation	[49]
	*b1*(booster 1)	Spontaneous	Reduced pigmentation	[50]
	*pl1* (purple plant 1)	Spontaneous	Reduced pigmentation	[51]
	*p1* (pericarp color 1)	Spontaneous	Reduced pigmentation	[32]
	*lpa1(low phytic acid1)*	Paramutagenic	High inorganic phosphate in seeds	[52]
*Linaria vulgaris*	*Lcyc* (Linaria cycliodea)	Spontaneous	Floral symmetry; dorsiventral flower axis	[38]
*Solanum lycopersicum*	*CNR* (Colorless non-ripening)	Spontaneous	Normal fruit ripening	[39]
	*VTE3* (Vitamin E)	Spontaneous	Tocopherol accumulation in fruit	[40]
*Oryza sativa*	*D1* (Drawf1)	Spontaneous	Dwarf	[53]
	*SPL14* (Squamosa Promoter binding protein-Like)	Spontaneous	Panicle branching and higher grain yield	[54]
	*FIE1* (*Fertilization-Independent Endosperm 1)*	Spontaneous	Dwarf	[55]
	*RAV6 [Related to Abscisic Acid Insensitive 3 (ABI3)/Viviparous1 (VP1) 6]*	Spontaneous	Larger lamina inclination and smaller grain size	[56]
	*AK1* (*Adenylate Kinase 1*)	Spontaneous	Defects in photosynthetic capacity	[57]
	*ESP (Epigenetic Short Panicle)*	Spontaneous	Short panicle	[58]
*Elaeis guineensis*	*DEF1 (DEFICIENS)*	Spontaneous	Mantled fruit	[59]
*Brassica rapa*	*SP11/SCR (S locus protein 11/S locus cystein rich)*	Trans-acting (small RNAs)	Self-incompatibility	[42]
*Cucumis melo*	*CmWIP1 (WASP/N-WASP-interacting protein 1)*	Transposon Insertion	Sex determination	[41]
